# Enhancing the productivity and proliferation of CHO-K1 cells by oncoprotein YAP (Yes-associated protein)

**DOI:** 10.1007/s00253-024-13122-5

**Published:** 2024-04-04

**Authors:** Farnaz Roshanmehr, Shahriyar Abdoli, Zahra Bazi, Maryam Jari, Majid Shahbazi

**Affiliations:** 1 Medical Cellular & Molecular Research Center, Golestan University of Medical Sciences, Gorgan, Iran; 2 Department of Medical Biotechnology, School of Advanced Technologies in Medicine, Golestan University of Medical Sciences, Gorgan, Iran; 3 Arya Tina Gene (ATG), Biopharmaceutical Company, Gorgan, Iran

**Keywords:** Chinese hamster ovary (CHO) cells, YAP (Yes-associated protein), Phospho-mutant YAP5SA, Productivity, Hippo pathway

## Abstract

**Abstract:**

CHO cells are extensively employed in biological drug industry to manufacture therapeutic proteins. Nevertheless, production of biopharmaceuticals faces obstacles such as limited growth and inadequate productivity. Employing host cell engineering techniques for CHO cells serves as a valuable approach to address the constraints encountered in biologics manufacturing. Despite advancements, most techniques focus on specific genes to address individual cellular challenges. The significance of YAP, transcriptional co-activator, cannot be overstated due to its involvement in regulating organ size and tumor formation. YAP’s influence extends to various cellular processes and is regulated by kinase cascade in the Hippo pathway, which phosphorylates serine residues in specific LATS recognition motifs. Activation of YAP has been observed to impact both the size and quantity of cells. This research investigates the effects of YAP5SA on proliferation, apoptosis, and productivity in CHO-K1 cells. YAP5SA, with mutations in all five LATS-target sites, is selected for its heightened activity and resistance to repression through the Hippo-LATS1/2 kinase signaling pathway. Plasmid harboring YAP5SA was transfected into EPO-CHO and the influence of YAP5SA overexpression was investigated. According to our findings, transfection of EPO-CHO cells with YAP5SA exhibited a substantial enhancement in CHO cell productivity, resulting in a 3-fold increase in total protein and EPO, as well as a 1.5-fold increase in specific productivity. Additionally, it significantly contributes in augmenting viability, size, and proliferation. Overall, the findings of this study exemplify the potential of utilizing YAP5SA to impact particular cellular mechanisms, thereby presenting an avenue for customizing cells to fulfill production demands.

**Key points:**

*• YAP5SA in CHO cells boosts growth, reduces apoptosis, and significantly improves productivity.*

*• YAP5SA regulates genes involved in proliferation, survival, and mTOR activation.*

*• YAP5SA increases productivity by improving cell cycle, c-MYC expression, and mTOR pathway.*

**Supplementary Information:**

The online version contains supplementary material available at 10.1007/s00253-024-13122-5.

## Introduction

Therapeutic protein drugs have become an essential element within the healthcare industry and have brought about a significant transformation in the range of treatment choices available for various diseases (Lagassé et al. 2017). They are gaining more significance within the pharmaceutical industry (Bhatwa et al. 2021; Lewis et al. 2013). Producing therapeutic proteins through recombinant techniques is crucial in the global economy and providing advanced medical care (Jadhav et al. 2014; O’Flaherty et al. 2020). The initiation of mammalian cell culture as the fundamental method for biopharmaceutical manufacturing in the industry was triggered by the approval of the human tissue plasminogen activator (Genentech, USA) by the FDA in 1986, which was the first therapeutic protein obtained from recombinant mammalian cells (Kim et al. 2012; Lee et al. 2015). During this specific timeframe, mammalian cells remain the predominant choice for expression systems, with a majority (107 out of 159, or 67%) of the approved products produced using recombinant methods in cell-based systems originating from mammalian cells. Chinese hamster ovary (CHO) cells, the most frequently utilized mammalian cell system, were responsible for generating 95 out of the 107 individual products produced in mammalian systems (accounting for 89% of the total). These findings demonstrate the recognized advantages of the CHO cell production platform with other mammalian systems such as NS0 mouse myeloma cells (seven products), baby hamster kidney (BHK), human embryonic kidney (HEK), sp2/0 mouse myeloma cells, and PER C6 immortalized primary human embryonic retinal cells (each contributing to 1 product) also being utilized to a lesser extent (Walsh and Walsh 2022). Indeed, CHO cell lines are the prevailing mammalian cell factories utilized to generate therapeutic proteins (Hamdi et al. 2020; Klanert et al. 2016). CHO cells possess various features that render them well-suited for generating recombinant human proteins, particularly in clinical settings (Mauro 2018). These features encompass the ability to undergo genetic alterations in a flexible manner (Tihanyi and Nyitray 2020), a lengthy track record of regulatory approval in biopharmaceutical production (Almo and Love 2014; Lewis et al. 2013; Nguyen and Zimmer 2023), and the potential for simplified approval processes from regulatory agencies such as the FDA for marketing therapeutic proteins (Kim et al. 2012). Furthermore, CHO cells can conduct post-translational modifications (PTM) on proteins derived from human sources (Pieper et al. 2017). This is significant due to the direct correlation between the effectiveness and immunogenicity of recombinant proteins and their PTM (Fischer et al. 2015; Xu et al. 2011). Moreover, CHO cells can be readily adjusted to thrive in suspension, in the absence of serum, and at increased levels of cellular concentrations (Hackl et al. 2014; Zucchelli et al. 2016). It has been shown that CHO cells serve as safe hosts for biologics production because they possess the capacity to fend off human viral infections. This is due to the fact that CHO cells do not typically express the genes responsible for viral entry, including cell surface adhesion molecules and plasma membrane proteins involved in viral recognition (Noh et al. 2013). Enhancing the production capabilities of CHO cells to generate substantial quantities of therapeutic proteins remains a significant hurdle within the biopharmaceutical sector (Latorre et al. 2023). Three primary strategies have been pursued to enhance the productivity of mammalian cell cultures: improving the expression vectors and transcription of the target gene, refining the composition of the culture media and cultivation processes, and engineering host cells (Dietmair et al. 2011; O’Flaherty et al. 2020).

**Fig. 1:**
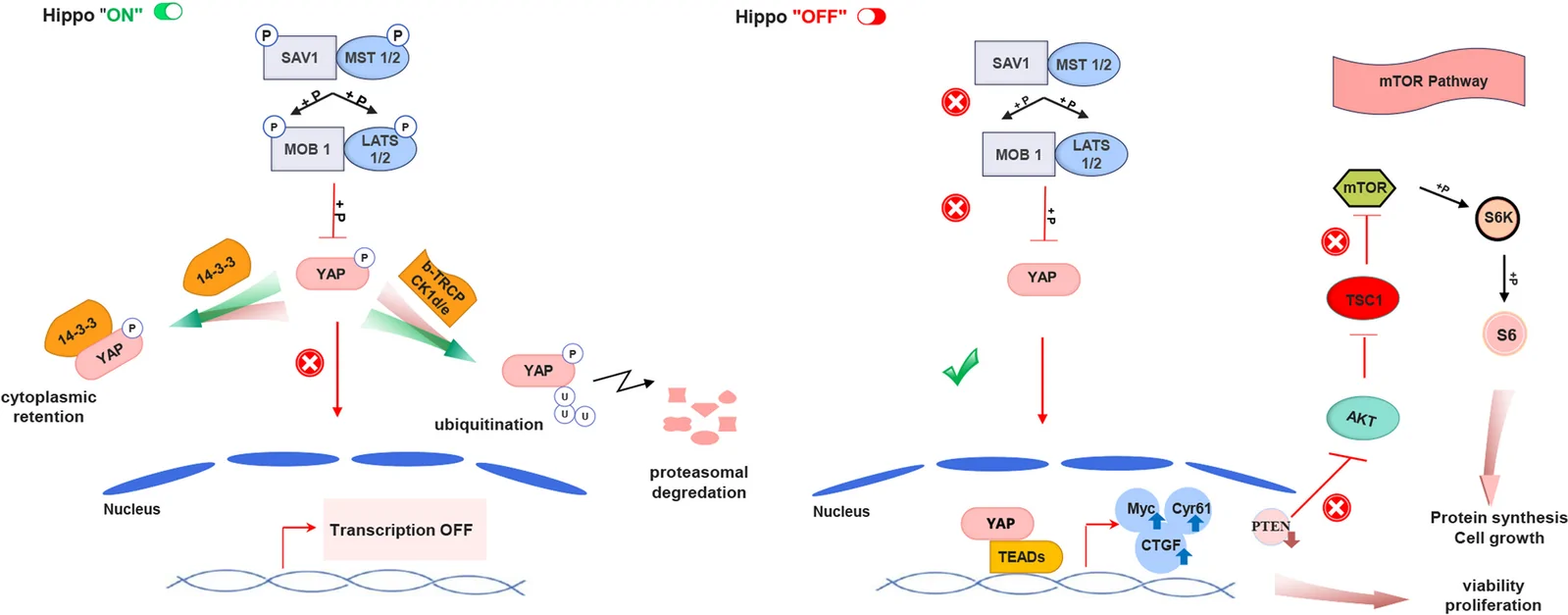
The proposed models depict the Hippo/YAP pathway and its interaction with the TOR pathway. Phosphorylation of YAP, recognized as a central element in the Hippo pathway, induces its confinement within the cytoplasm and subsequent breakdown through its interaction with 14–3-3 proteins and b-TrCP, respectively. Consequently, YAP can be restrained by spatial segregation from its nuclear target transcription factors, such as TEAD (left). When the Hippo pathway becomes deactivated, YAP undergoes translocation into the nucleus. As a transcriptional co-activator, YAP interacts with the TEAD transcription factor to promote the transcription of genes associated with cell proliferation and survival, such as *c-MYC*, *CTGF*, and *CYR61*. Additionally, YAP can establish interconnections with the PI3K/mTOR pathway by suppressing *PTEN*, an upstream inhibitor of mTOR (right)

A significant portion of cell engineering efforts has concentrated on the manipulation of genes. However, it is evident that when modifying cells to enhance their resilience in producing recombinant proteins and monoclonal antibodies, it is essential to consider the overall impact of modifying one or multiple genes throughout various cellular pathways (Lim et al. 2010). Cellular proliferation and apoptosis are interconnected and contrasting evolutionary processes that play crucial roles in the progression and maturation of organisms. The disruption of either cellular proliferation or apoptosis serves as a catalyst for the development and progression of cancer (Loftus et al. 2022). Within this framework, the Hippo pathway serves as a widely preserved tumor suppressor pathway that governs cell proliferation, apoptosis, and the self-renewal of stem cells. Its primary role is to regulate the number and size of cells within organs (Kedan et al. 2018). Dysregulation of this pathway is linked to various stages of cancer development and progression (Luo et al. 2023; Mokhtari et al. 2023; Xiao and Dong 2021). When the Hippo signaling pathway undergoes dysregulation, the nuclear translocation of its downstream mediator YAP occurs, leading to its interaction with transcription factors, including TEAD (TEA domain) and other factors. This interaction leads to enhanced transcriptional activation of genes associated with cell proliferation and survival (Park et al. 2016). YAP is primarily recognized for its role in promoting unrestricted cell growth and proliferation during the progression of tumors (Han et al. 2020; Park et al. 2016). It is noteworthy that the activation of the Hippo pathway functions as a “restraint” on transcription (Sugihara et al. 2018). The Hippo pathway comprises an upstream cascade of serine kinases (MST1/2 and LATS1/2) that govern the activity of co-transcriptional activators, such as YAP (Fu et al. 2022). YAP contains five HXRXXS motifs, which are phosphorylation sites for LATS kinases (Zhao et al. 2010). Phosphorylation of these serine residues on YAP leads to its sequestration within the cytoplasm due to its interactions with the protein 14–3-3. This restricts YAP’s role as a transcriptional co-activator (Sugihara et al. 2018). Concomitant with its sequestration in the cytoplasm, there is an augmentation in the ubiquitination of YAP and subsequent degradation through the proteasome (Kedan et al. 2018). At the same time, YAP, which has not undergone phosphorylation, relocates to the nucleus. There, it engages with transcription factors from the TEAD family and acts as a co-activator for transcription. Significant genes targeted by YAP are those associated with cell growth and proliferation, such as Connective Tissue Growth Factor (*CTGF*), Cysteine-Rich Angiogenic Inducer 61 (*CYR61*), and MYC proto-oncogene (*MYC*) (Yamaguchi and Taouk 2020). Besides, YAP can modulate gene expression and signaling pathways associated with cell growth in various cell types. Intriguingly, emerging evidence suggests a mutual influence between the Hippo and mTOR pathways in regulating cellular growth (Gan et al. 2020; Goodman et al. 2015; Park et al. 2016; Xu et al. 2021). Specifically, YAP facilitates communication between the Hippo and PI3K-TOR pathways by inhibiting PTEN, which is a suppressor of mTOR, thereby influencing their interplay (Xu et al. 2021) (Fig. 1).

There is a need for more knowledge regarding the role of the YAP protein in CHO cells. Therefore, in this study, we first aim to address this gap by developing modified CHO cell lines through the overexpression of YAP5SA, which is assumed to have constitutive activity. We then investigate the impact of overexpressing YAP5SA on regulating vital cellular processes in CHO cells, including growth, proliferation, viability, and productivity.

## Materials and methods

### Identifying phosphorylation sites of LATS in *Cricetulus griseus* YAP protein by alignment to conserved sequences

To identify and compare the LATS consensus sites across different mammalian species, including human, mouse, and Chinese hamster, the amino acid sequences of the YAP protein were aligned using COBALT. This alignment allowed for examining similarities and differences in the LATS consensus sites among the various species.

### Expression vector design

The cDNA sequence encoding the *Cricetulus griseus* YAP1 protein was acquired from the National Center for Biotechnology Information (NCBI) under the accession number XM_027415168.2. In order to generate a mutant form of YAP, YAP5SA, eight serine residues on all five Lats phosphorylation consensus sites have been mutated to alanine (S48A, S96A, S114A, S115A, S118A, S149A, S150A, and S367A). These sites are well-established targets of the Lats kinase, which phosphorylates YAP and subsequently triggers its cytoplasmic retention and degradation. By mutating these serine residues to alanine, we aimed to generate a YAP mutant (YAP5SA) that cannot be phosphorylated by Lats, thereby preventing its nuclear exclusion and degradation.

The expression of the YAP5SA protein was achieved using the CMV early enhancer/chicken β actin (CAG) promoter. The specified sequence was inserted into the piggyBAC plasmid, PB513B-1, and synthesized by Gene Scripts in China. The nucleotide sequence encoding the YAP5SA, which has been inserted into the PB513B-1 plasmid, is provided in Fig. S2 (Online Resource 1).

### Cell culture and transfection

The CHO-K1 cell line was obtained from the Pasteur Institute (Tehran, Iran). A stable erythropoietin-producing CHO-K1 cell line (EPO-CHO) had previously been developed and generated in our laboratory. This cell line was created by introducing a plasmid into CHO-K1 cells. This plasmid contains the human erythropoietin (hEPO) gene (accession number: M11319) and the puromycin resistance gene, which acts as a marker for selection and survival of transfected cells during the establishment of the stably transfected cell lines. To establish a stable CHO cell line capable of producing hEPO, a selective pressure of 5 µg/ml puromycin was employed. These cell lines were cultivated in a medium composed of DMEM/F-12 (GIBCO, Life Technologies Inc., USA), supplemented with 10% FBS and 50 µg/mL penicillin/streptomycin (P/S), at 37 °C, 5% CO_2_, and 95% humidity.

In order to assess the effects of YAP5SA overexpression on various bioprocess relevant parameters, including viable cell density, product titer, and specific productivity, the construct encoding the YAP5SA protein (OE-YAP5SA plasmid) was introduced into the EPO-CHO cell line. Simultaneously, a separate group of cells was transfected with the PB513B-1 vector, which solely consists of the vector backbone without the transgene (blank construct). This transfected cell group served as a Mock control for comparison purposes. The transfection was carried out using ScreenFect^TM^A reagent (FUJIFILM, Japan) as the transfection agent, following the manufacturer’s protocol. An additional control group consisting of EPO-CHO cells that were not subjected to transfection (referred to as NT) was included to ensure thorough comparison. This control group functions as a baseline to understand the fundamental properties of the host cells, including productivity, viability, and growth, without any genetic alterations or transfection. It offers insights into the inherent behavior of the parental CHO cells.

### Total RNA extraction, reverse transcription, and real-time PCR

The extraction of total RNAs was carried out using the TRIzol reagent (Biobasic, Canada) following the instructions provided by the manufacturer. DNase treatment was applied. Reverse transcription was performed using random hexamer primers and the cDNA Synthesis Kit (Yekta Tajhiz Azma Co.; Tehran, Iran) following the manufacturer’s guidelines. Quantitative PCR (qPCR) was conducted using the SYBR Green qPCR master mix (Yekta Tajhiz Azma Co; Tehran, Iran). The primers utilized are provided in Table 1. The LineGeneK Fluorescence qPCR machine (Hangzhou Bioer Technology, China) was used for the qPCR assay. β-Actin (ACTB) was utilized as a control. Each biological replicate was subjected to three technical replicates for measurement.

**Table 1:** Utilized primer sequences in reverse transcription-polymerase chain reaction analysis

Gene symbol	Forward (5′-3′)	Reverse (5′-3′)
*YAP1*	TGCTCTCCCAACTGAATGTC	CTTGCTCCCATCCATCAGG
*CCN1*	TGAAGAGGCTTCCTGTCTTTG	ATTCTGGGTTGTCATTGGTAAC
*CCN2*	ACCTGGAAGAGAACATTAAGAAGG	GTCAGGACATTTGAACTCCAC
*MTOR*	AGGTGTGGTTTGACCGAAGA	CAGGTTGGATGGGTGTCTGT
*MYC*	CACGTCTCCACTCATCAGCA	TCGTTTCTCCTCTGGCGTTC
*RPS6KB1*	ATCATGCTCAATCACCAAGGTC	AACTCCACCAATCCACAGCA
*BAX*	TCCGTCTACCAAGAAGTTGAG	GTATCCACATTAGCAATCATCCTC
*PTEN*	CCAGTCAGAGACGCTATGTG	GACCACAAACTGAGGATTGC
*TSC1*	ACCGTTCAGCAGATGTCACC	GGCTCCAGTGAGTAGCTTGC
*ACTB*	GCCTTCCTTCCTGGGTATG	CTTGATCTTCATGGTGCTGG

*YAP1*, Yes-associated protein 1; *CCN1*, cysteine rich angiogenic inducer 61 (CYR61); *CCN2*, connective tissue growth factor (CTGF); *MTOR*, mammalian target of rapamycin; *MYC*, proto-oncogene C-Myc; *RPS6KB1*, ribosomal protein S6 kinase B1; *BAX*, BCL2-associated X; *PTEN*, phosphatase and tensin homolog; *TSC1*, TSC complex subunit 1; *ACTB*, actin beta

### Protein isolation, SDS-PAGE, and western immunoblotting

To validate the presence of YAP5SA protein in the transfected transgenic erythropoietin (EPO)-producing CHO cell line, SDS-PAGE electrophoresis was conducted, followed by subsequent western blotting analysis. The cells were lysed using 200 μl RIPPA buffer supplemented with a protease inhibitor cocktail and PMSF. Following a 15-min incubation on ice, the cellular debris was removed by centrifugation at 12,000 × *g* at 4 °C for 20 min. The protein content in the supernatant was determined using a BCA protein assay kit (DNA Biotech, Iran). Subsequently, a loading buffer was added to the supernatant. The exact quantity of total protein per lane underwent separation using 10% sodium dodecyl sulfate (SDS) polyacrylamide gel electrophoresis and transferred onto a nitrocellulose membrane (WhatmanGmbH; Germany). After blocking with a 3% skim milk solution in PBS-T for 2 h at room temperature, the membranes were exposed to primary antibodies and incubated overnight at 4 °C. These antibodies included Anti-YAP (rabbit, diluted at 1:500, LifeSpan Biosciences # LC-C331201/214837) and Anti β-Actin (mouse, diluted at 1:5000, Mellat Biotechnology Development # MB6276). After a washing step, the blots were exposed to the appropriate HRP-labeled secondary antibodies (anti-rabbit antibody (goat, diluted at 1:1000, sigma #A6154) and anti-mouse antibody (sheep, diluted at 1:2000, Mellat Biotechnology Development # MB6808)) and incubated for 2 h at room temperature. The blots were subsequently developed using DAB (3,3′-Diaminobenzidine). Beta-actin was employed as a control for loading.

### Cell counts

The determination of viable cell count and alteration in cell proliferation rate was performed using the Trypan blue exclusion method with a hemocytometer (Neubauer, Germany). This method allowed for differentiation between viable and nonviable cells based on the uptake of the staining agent.

### Viability assessment

To evaluate the impact of YAP5SA overexpression on cell viability, EPO-CHO cells were seeded and grown in 96-well plates until they reached approximately 50% confluency. After 24, 48, and 72 h of transfection, a colorimetric cell viability assay was performed using the MTS proliferation assay kit (Promega, USA). To perform the assay, combining the MTS and PMS components beforehand is necessary, followed by an incubation period of 1 to 4 h. In brief, 100 µl of supernatant was removed from each well, and then 100 µl of fresh medium and 20 µl of the MTS-PMS reagent were added to allow for colorimetric evaluation of viable cells. The plates were subsequently read using an ELISA plate reader (Biotek; Germany), and the obtained data were documented.

### Assessment of apoptosis by Annexin/PE staining

The goal of this experiment was to determine the number of cells that underwent apoptosis, and it was carried out using the PE Annexin V Apoptosis Detection kit with 7-AAD (BioLegend, San Diego, California, USA). In brief, CHO cells were initially cultured in 6-well plates and subsequently transfected with OE-YAP5SA or PB plasmid as a control. The cells were then detached using trypsin, washed with ice-cold PBS, centrifuged, and re-suspended in 100 μL of binding buffer. To each tube, 5 μL of Annexin V and 10 μL of 7-AAD were added. The mixture was incubated at room temperature for 15 min. After incubation, 400 μL of binding buffer was added to each tube, and the samples were analyzed using a BD FACSCalibur flow cytometer (BD Biosciences, USA). The PE and 7-AAD fluorochromes were excited by 488-nm laser with an emission peak at 578 nm and 650 nm, respectively. This assay was performed twice for accuracy. The flow cytometry data was analyzed by FlowJo™ v10.5.3 Software (BD Life Sciences).

### Cell cycle analysis

In order to assess changes in the distribution of cell-cycle phases following transfection with the OE-YAP5SA plasmid, cells were subjected to propidium iodide (PI) (BioLegend, San Diego, California, USA) staining to evaluate their DNA content. To put it briefly, the cells were gathered 48 h after transfection, fixed using 70% ethanol, and dyed with PI. Subsequently, cell cycle analysis was conducted utilizing a BD FACSCalibur flow cytometer (BD Biosciences, USA). The excitation of PI occurred using a 488-nm laser, leading to an emission peak of approximately 610 nm. The obtained data was then analyzed utilizing ModFit LT software (Verity Software House, Topsham, ME, USA).

### Analysis of cell size

EPO-CHO cells were initially placed in a 6-well plate and then transfected with prenominated plasmids. Following a 48-h incubation period, the cells were washed with 5 mL of PBS and subsequently detached using trypsin. The cells that were gathered went through two rounds of washing with 10 mL of ice-cold PBS, where each round consisted of centrifugation at a force of 2000 g for 5 min. Subsequently, the cells were suspended again in 500 mL of PBS. Their forward light scatter (Fsc), which signifies cell size, was documented by flow cytometry.

### Immunoassay for the detection and quantification of the erythropoietin protein

The stable EPO-CHO cells were transfected with OE-YAP5SA or control constructs. Following a 48-h incubation period, the supernatant from the cell culture was gathered. The quantity of secreted EPO was then assessed using the EPN kit (L2KEPN2) on the Siemens Immulite® 2000 XPi immunoassay system, an automated analyzer that utilizes chemiluminescent immunoassay technology. This system uses polystyrene beads which are coated with assay-specific antibody or antigen as the solid phase. Beads are placed in specially designed reaction tubes which is used as vessel for incubation, washing, and signal processing. Following the incubation with an HRP-labeled reagent, the reaction mixture is separated from the bead by subjecting the reaction tube to high-speed centrifugation along its vertical axis. The liquid is moved to a sump chamber that is connected to the bead/tube wash station. Within a matter of seconds, as many as four distinct washes take place, enabling the consecutive handling of reaction tubes with consistent timing. The bead stays inside the reaction tube with minimal remaining label that is not bound. The attached label is measured by utilizing an enhanced chemiluminescence (ECL) substrate. When this substrate reacts with the HRP label bound to the bead, it generates light with a maximum wavelength of 425 nm. The intensity of the emitted light is directly proportional to the quantity of the analyte that was originally present in the sample. A photomultiplier tube (PMT) is utilized to detect the emitted light, and the measurements are computed for each sample.

### Statistical analysis

The experiments were conducted in duplicate or triplicate biological replicates. All values are represented as the mean ± standard deviation (S.D.). To compare differences between any two groups, a two-tailed unpaired *t*-test was utilized. The significance levels were clarified as P* ≤ 0.05, P** ≤ 0.01, P*** ≤ 0.001, and P**** ≤ 0.0001. The data was imported into Prism 8.0 software (GraphPad Software Inc., La Jolla, CA, USA) for visualization, fitting, and statistical analysis. Statistical analysis between groups was assessed using one-way or two-way ANOVA.

## Results

### The intact phosphorylation motif of LATS is present in the YAP protein of *Cricetulus griseus*

The analysis of the YAP protein sequences from various mammalian species, including human (hYAP), mouse (mYAP), and Chinese hamster (chYAP), revealed that the consensus phosphorylation motif of LATS, which is HXRXXS, remains completely unchanged in the sequence of chYAP (Table 2). This indicates that Chinese hamster possesses a conserved LATS consensus site within its YAP protein sequence. The identification of this intact phosphorylation motif suggests the potential for LATS-mediated regulation of YAP in *Cricetulus griseus*, similar to other mammalian species. Fig. S1 (Online Resource 1) displays the complete alignment of the YAP protein sequences.

**Table 2:**
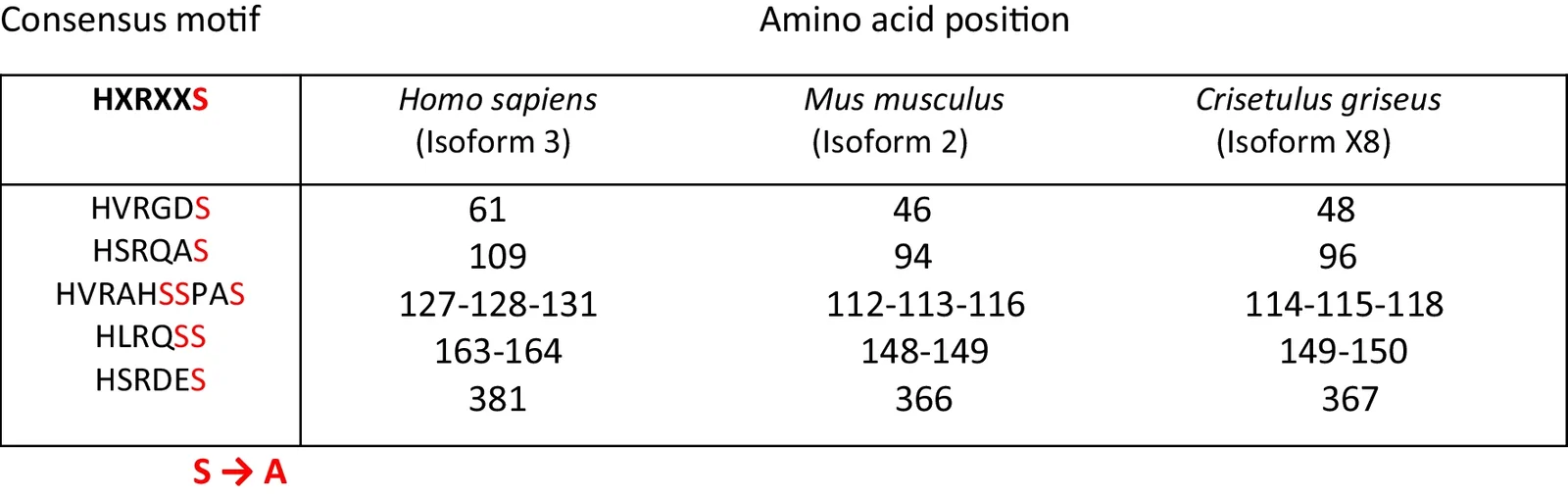
LATS phosphorylation sites: Conversion of eight serine residues to alanine (S → A) within five LATS consensus sites

### The YAP5SA construct exhibited elevated levels of YAP expression, both at the mRNA and protein levels

**Fig. 2:**
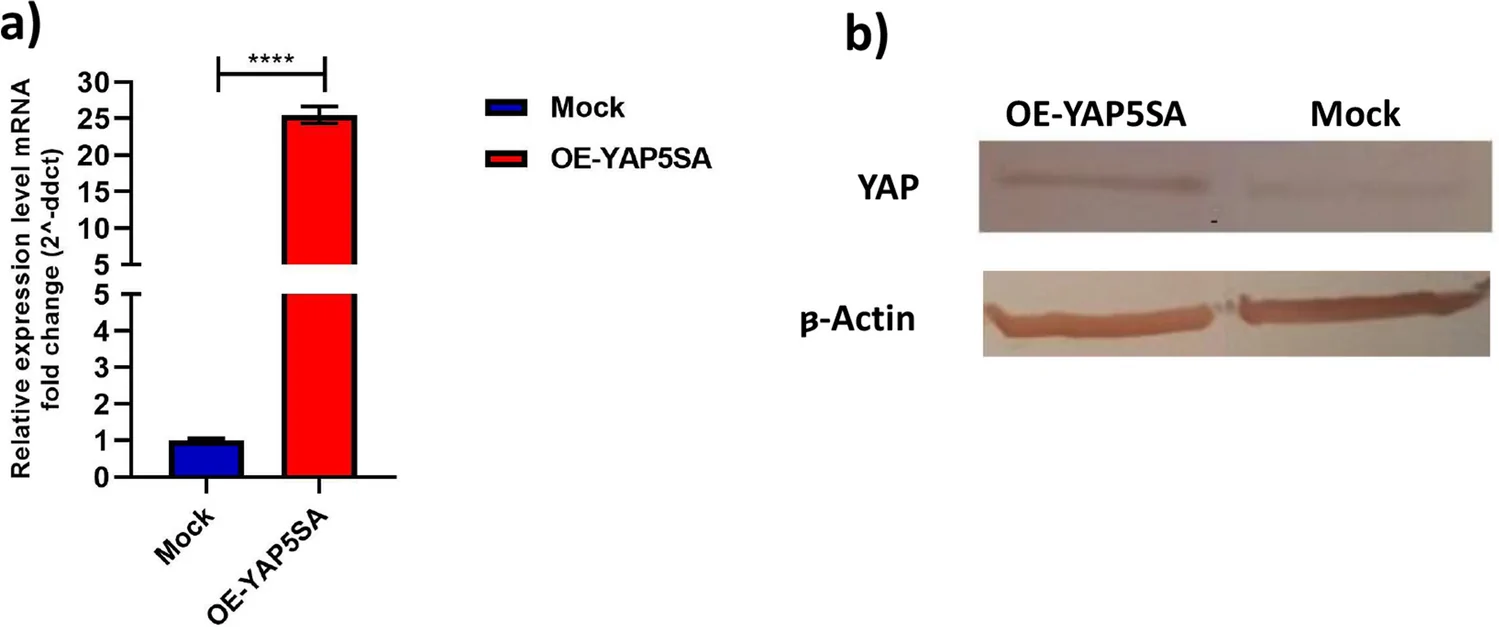
**a** Real-time analysis of YAP mRNA levels following overexpression of mutant YAP compared to control EPO-CHO cells. **b** Western immunoassay of biological samples with YAP protein overexpression observed in the OE-YAP5SA CHO cell line. An equal quantity of proteins was isolated on a SDS–polyacrylamide gel, transferred onto a nitrocellulose membrane, and subjected to immune staining using an anti-YAP antibody. β-Actin was utilized to ensure consistent protein loading. Both real-time PCR and western immunoblot analysis validated the successful upregulation of YAP expression

The results obtained from quantitative PCR (qPCR) demonstrated a significant increase in YAP expression within cells transfected with the YAP5SA construct in contrast to the control cohort. Statistical analysis of the qPCR data revealed that the YAP level in OE-YAP5SA cells was approximately 25-fold higher than that within the control cell groups (*P*-value < 0.0001) (Fig. 2a). Furthermore, a western blot immunoassay was conducted to validate the overexpression of YAP protein in OE-YAP5SA cells (Fig. 2b). Collectively, these findings indicate that the overexpression of YAP5SA in the transfected cells was successful.

### The expression levels of YAP target genes and the genes associated with the mTOR pathway exhibited a substantial alteration following the overexpression of YAP5SA in EPO-CHO cells

**Fig. 3:**
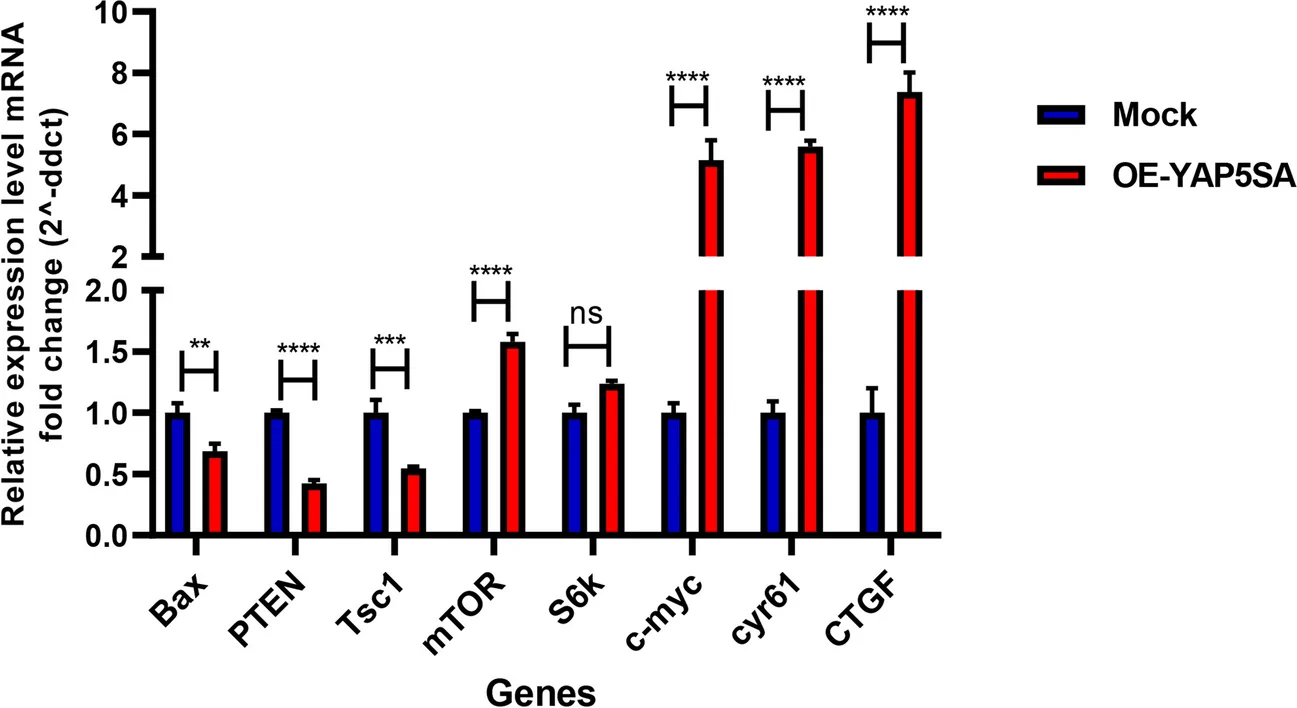
Real-time PCR analysis of samples obtained from OE-YAP5SA and control EPO-CHO cells. To normalize the data, mRNA levels of β-actin were used, and the results were presented as the average fold change in expression. Error bars represent the standard error of the mean (S.E.M) of triplicate determinations. (* p < 0.05; ** *p* < 0.01; *** *p* < 0.001; **** *p* < 0.0001; ns, not significant)

Dephosphorylated YAP interacts with transcription factors, particularly those from the TEAD family, leading to the activation of specific genes. This process plays a crucial role in YAP-induced cell proliferation and growth promotion. Therefore, assessing the expression levels of established YAP target genes such as *CTGF*, *CYR61*, and *MYC* proto-oncogene is a critical method for evaluating the activity of the Hippo pathway. These genes are essential for mediating YAP’s effects on cell growth and proliferation. The analysis of gene expression using qRT-PCR demonstrated a significant elevation in the expression of target genes upon the introduction of the OE-YAP5SA construct (*P*-value < 0.0001) (Fig. 3).

Considering the involvement of YAP in modulating the AKT/mTOR pathway in regulating cell size and tissue growth, we aimed to investigate the impact of YAP5SA on the mTOR pathway in EPO-CHO cells. Consequently, we investigated the expression of genes associated with mTOR signaling, specifically *PTEN*, *TSC1*, *S6K*, and *mTOR*. The overexpression of YAP5SA resulted in the downregulation of genes that have inhibitory effects on mTOR activation, such as *PTEN* and *TSC1* (*P*-value < 0.0001 and *P*-value < 0.001, respectively). Furthermore, YAP overexpression resulted in the upregulation of *mTOR* expression (*P*-value < 0.0001), whereas no remarkable changes in the expression of *S6K* were observed (Fig. 3).

Moreover, there is evidence supporting the involvement of YAP in regulating *BAX*, which is a transcriptional target of p73. As illustrated in Fig. 3, the upregulation of YAP5SA leads to a significant downregulation in the expression of *BAX* (*P*-value < 0.01).

### Overexpression of YAP5SA affected the viability and proliferation of EPO-CHO cells

Cell productivity is influenced by two crucial factors: cell proliferation and cell viability. Viable cell density (VCD) was determined using trypan blue staining and viability was assessed using the MTS. The results are presented as the mean ± SD.

**Fig. 4:**
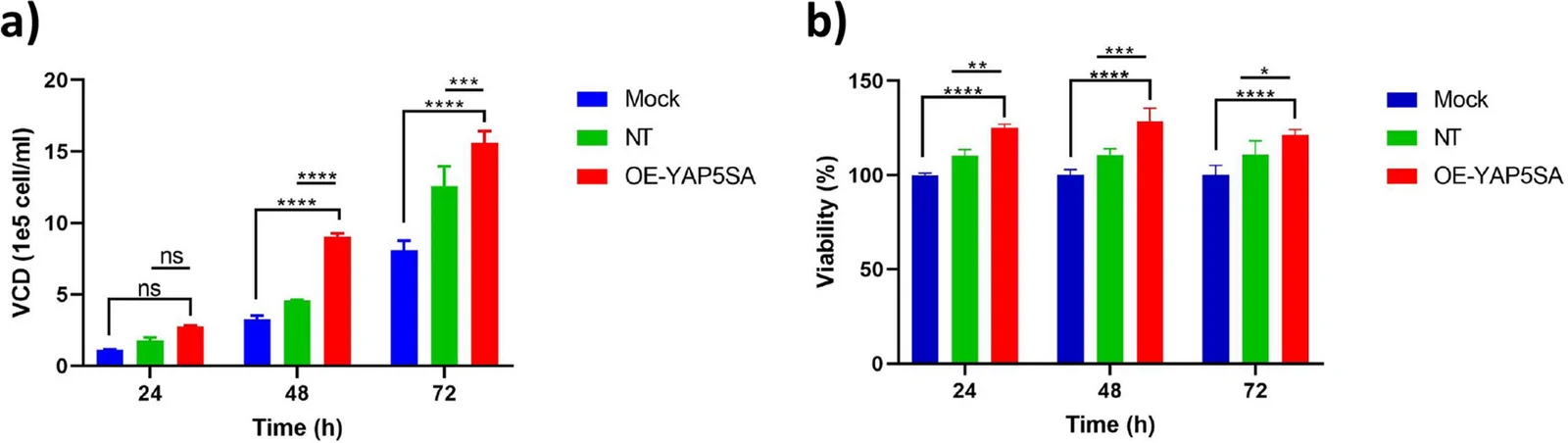
YAP5SA-induced cell growth and viability in CHO cells using the **a** Trypan blue and **b** MTS assays. The cells were seeded on 96-well plates (7 × 10^3 cells/well) and transfected with overexpressing YAP5SA and control plasmids. Following an incubation period of 1, 2, and 3 days, cell viability was determined using both assays. Error bars represent the standard deviation (S.D)

The outcomes of the VCD experiment reveal that after 24 h, the Mock and NT groups exhibited a VCD of 1.12 × 10^5^ ± 0.035 and 1.79 × 10^5^ ± 0.19 cells/ml, respectively, while the VCD in the OE-YAP5SA group increased by almost 2-fold, although this increase was not statistically significant. Following 48 h, the Mock and NT groups displayed a VCD of 3.25 × 10^5^ ± 0.29 and 4.55 × 10^5^ ± 0.06 cells/ml, respectively, whereas the OE-YAP5SA group exhibited a significantly higher VCD of 9.05 × 10^5^ ± 0.21 cells/ml. Importantly, after 72 h, the VCD of the Mock and NT groups reached 8.11 × 10^5^ ± 0.66 and 12.56 × 10^5^ ± 1.38 cells/ml, respectively, while the VCD of the OE-YAP5SA group peaked at 15.6 × 10^5^ ± 0.84 cells/ml. These findings indicate a time-dependent increase in VCD for all groups. Notably, the OE-YAP5SA group consistently demonstrated a significantly higher VCD compared to both the Mock and NT control groups at all time points, except at 24 h (*p* > 0.05) (Fig. 4a).

The outcomes of the MTS assay demonstrated that the overexpression of YAP5SA after a duration of 24 h resulted in an increase in the viability of CHO cells by approximately 25% and 15% compared to the Mock and NT groups, respectively. However, this elevation did not exhibit a significant change in subsequent time intervals (Fig. 4b). In general, these findings indicate that OE-YAP5SA exerts a considerable impact on promoting cell proliferation, enhancing viable cell density, and improving cell viability, particularly at later time points.

### The overexpression of YAP5SA resulted in a decrease in apoptosis

To validate the results obtained from the MTS assay, the level of apoptosis was assessed in the experimental group (OE-YAP5SA) as well as the control groups (Mock and NT). Apoptotic cells were detected using annexin V-PE and 7-AAD flow cytometry, and the results were reported as the mean ± SD of apoptotic cells.

**Fig. 5:**
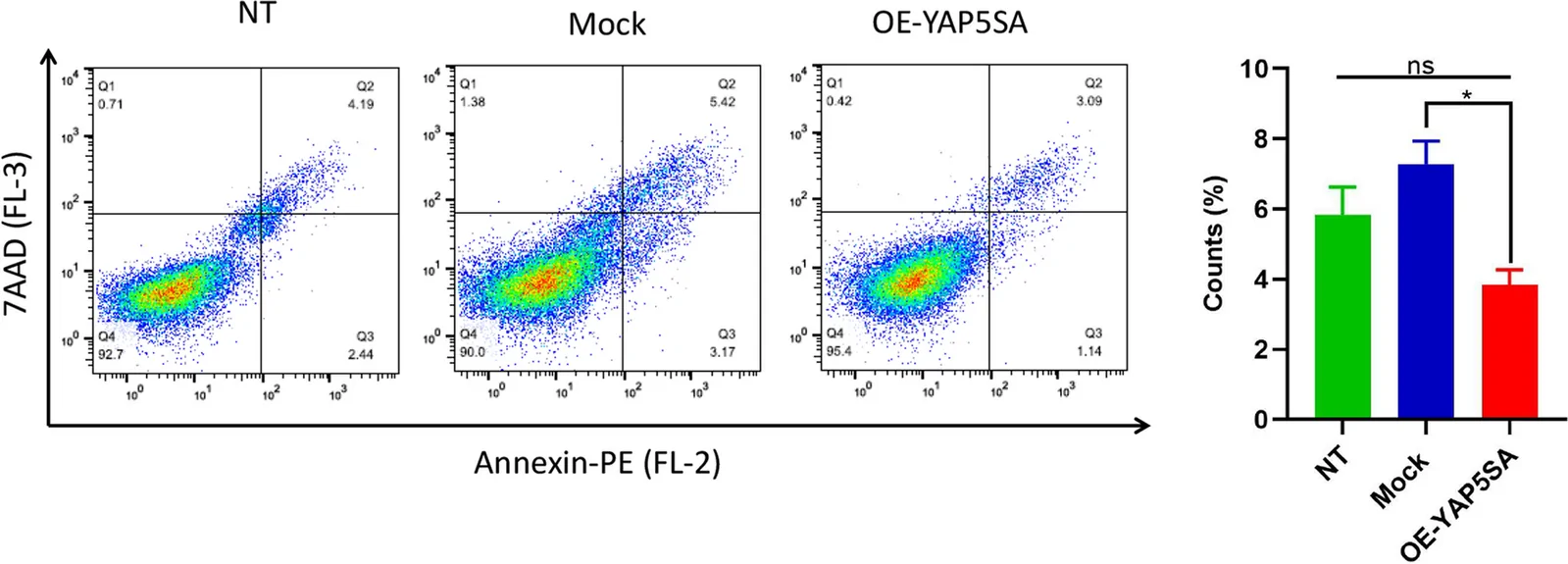
Apoptosis analysis of OE-YAP5SA EPO-CHO compared to control cells: We examined the cells using Annexin V-PE and 7-AAD staining. Error bars represent the standard deviation (S.D)

The Mock group exhibited a mean percentage of apoptotic cells of 7.27% ± 1.13% (mean ± SD), while the NT group showed a mean percentage of apoptotic cells of 5.83% ± 1.12% (mean ± SD). In comparison, the OE-YAP5SA group showed a significantly lower mean percentage of apoptotic cells, measuring 3.83% ± 0.77% (mean ± SD). The findings from this assay, presented in Fig. 5, reveal that the apoptosis rate was reduced when there was an overexpression of YAP5SA. These results indicate that the introduction of OE-YAP5SA in EPO-CHO cells leads to a reduction in apoptosis compared to the Mock control cells (*P*-value < 0.05).

### Enhanced expression of YAP5SA promotes the transition from the G1-to-S phase

As cell proliferation is closely associated with cell-cycle advancement, we compared the cell-cycle profiles of cell lines overexpressing YAP5SA and the control EPO-CHO cells. Flow cytometry was employed to assess the proportion of cells in various phases of the cell cycle.

**Fig. 6:**
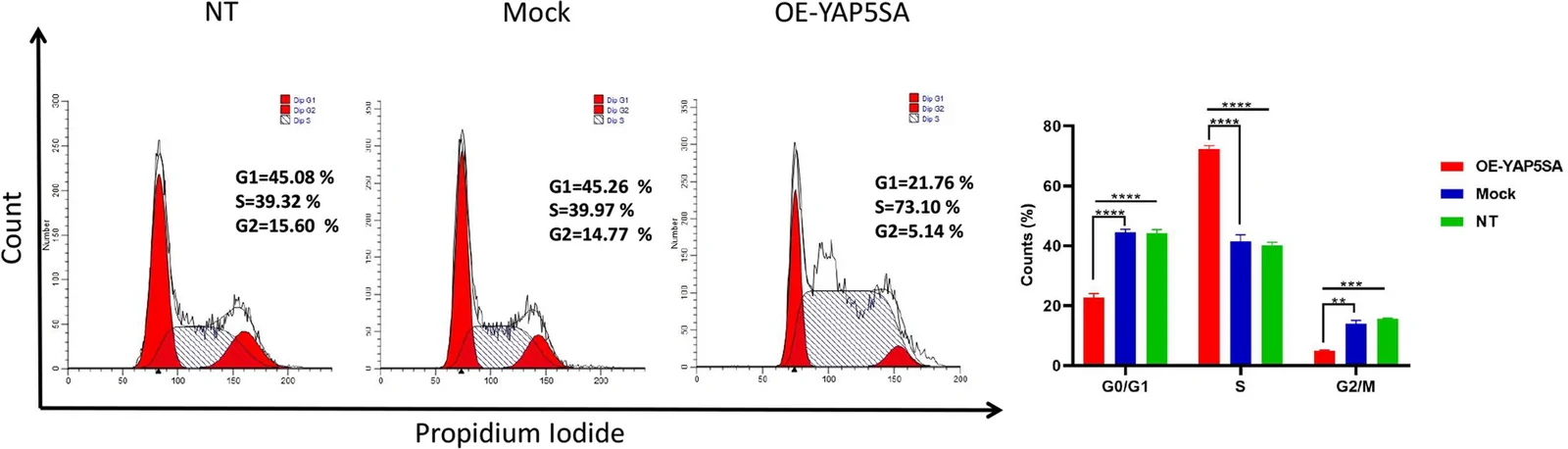
Cell cycle distribution determined by flow cytometry; cell count vs. fluorescence. After subjecting the cells to a 48-h treatment with the YAP5SA construct and a blank plasmid (control), they were dyed with PI and assessed for DNA content. A substantial increase in the S phase was observed in OE-YAP5SA cells compared to Mock and NT. The percentage of cells in each phase is indicated alongside each figure. Two-way ANOVA was employed to conduct statistical analysis. Error bars represent the standard deviation (S.D). (* *p* < 0.05; ** *p* < 0.01; *** *p* < 0.001; **** *p* < 0.0001)

The percentage of cells in the S-phase of the cell cycle is approximately twice as high in OE-YAP5SA cell lines compared to Mock control cells (*P*-value < 0.0001) (Fig. 6). Simultaneously, there was a decrease in the percentage of cells in the G0/G1 phase (*P*-value < 0.0001) and the G2/M phase (*P*-value < 0.01). This indicates that YAP5SA promotes the progression of the cell cycle by inducing the transition from G1 to the S phase. This discovery aligns with the observed patterns of proliferation, as depicted in Fig. 4.

### YAP5SA increases cell size

**Fig. 7:**
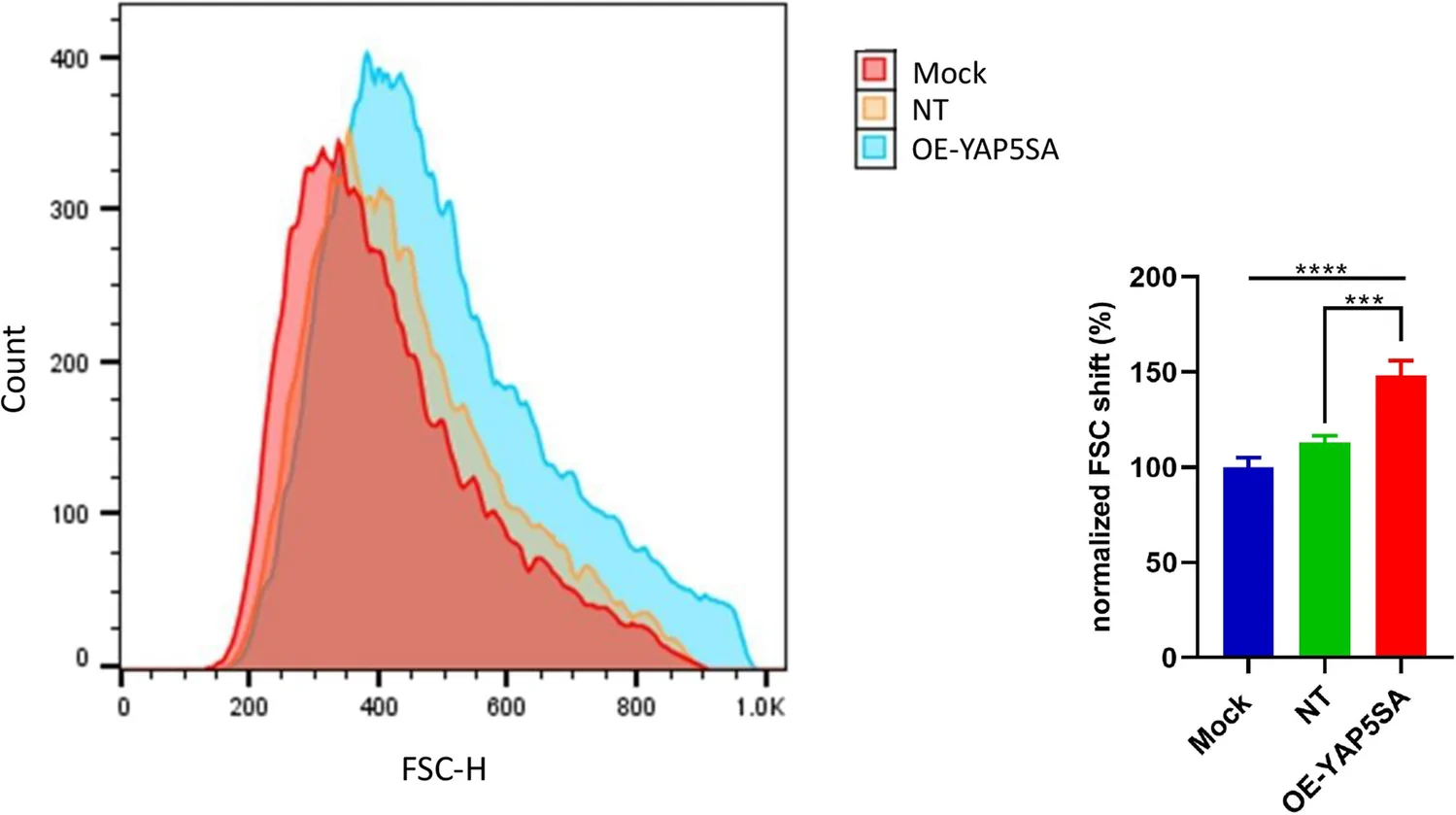
The impact of YAP5SA expression on cell size. Cell size profiles were obtained through flow cytometry-mediated analysis of the FSc (forward scatter). Error bars represent the standard deviation (S.D)

**Fig. 8:**
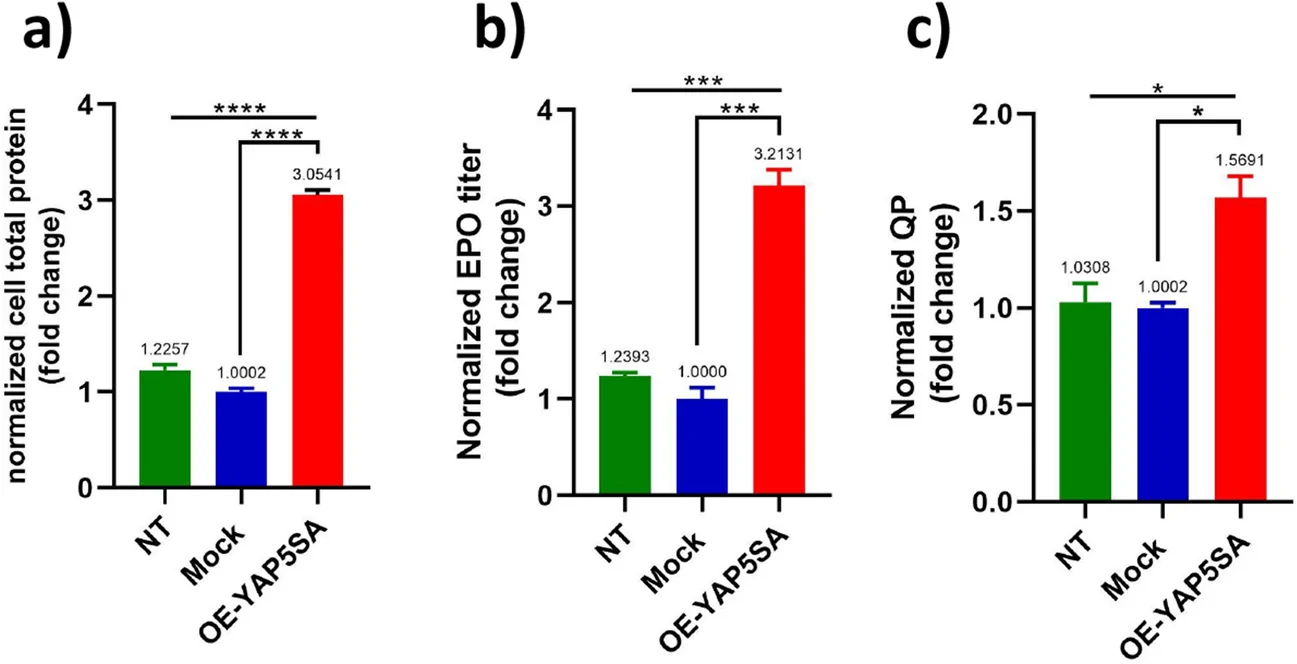
The protein production capability of YAP5SA-overexpressing EPO-CHO cell line. The impact of YAP5SA overexpression on the production of both total protein and EPO protein was assessed. The overexpression of YAP5SA led to a rise in total protein production in EPO-CHO cells (**a**). Additionally, it enhanced the volumetric production of EPO (**b**) and the specific productivity of recombinant EPO in EPO-CHO cells (**c**). Specific productivity was determined by calculating the EPO concentration in the supernatant and normalizing it to control cells. Error bars represent the standard deviation (S.D)

In order to assess the influence of YAP5SA overexpression on the growth of stable EPO-CHO-k1 cells, we performed a cell size analysis utilizing flow cytometry. Remarkably, the flow cytometry analysis unveiled a shift towards elevated forward scatter (FSc) values within the OE-YAP5SA cells, thereby signifying an augmentation in cell size compared to Mock and NT (*P*-value < 0.0001 and *P*-value < 0.001, respectively) (Fig. 7). The observed enhancement in specific productivity (QP) in OE-YAP5SA EPO- CHO cells (Fig. 8c) is primarily attributed to an increase in cell size.

### YAP5SA overexpression resulted in an enhancement of protein synthesis and an increase in erythropoietin production

Our interest was to investigate whether YAP5SA-transfected cells in stable EPO-CHO-K1 populations could impact the production of global and specific proteins, particularly EPO, compared to the control population. To explore this, we assessed the production capacity of the cells. After 48 h of transfection, the EPO concentration in the supernatant and the total protein content were measured. The outcomes presented in Fig. 8 exemplify that the YAP5SA overexpressing cells produced approximately three times more total protein than the EPO-CHO cells transfected with the blank construct and NT cells (*P*-value < 0.0001). The EPO production levels, measured in ug/mL, were determined for each sample, along with the EPO productivity per cell. The cells expressing YAP5SA exhibited higher specific production levels than the control cells. Notably, the overexpression of YAP5SA in EPO-CHO-K1 cells led to a minimum 3-fold rise in titer compared to control cells (*P*-value < 0.001), as well as a 1.5-fold increase in specific productivity (QP) (*P*-value < 0.05).

## Discussion

The primary driver of the highly conserved Hippo pathway is a protein called YAP (Xu et al. 2020). YAP plays a crucial role in stimulating cell growth and preventing cell death by activating specific transcription factors known as the TEAD family transcription factors. This activation leads to the expression of particular genes involved in cell proliferation while inhibiting apoptosis. The kinases LATS1/2, which act as negative regulators, directly add phosphate groups to specific serine residues on YAP in five consensuses HXRXXS motifs (Zhao et al. 2011). This process leads to YAP being confined within the cytoplasm or broken down through the ubiquitination pathway (Xu et al. 2020; Zhao et al. 2010). Hence, it is possible to hinder the activity of YAP by physically separating it from the transcription factors it targets within the nucleus. As shown, altering the serine phosphorylation site of the YAP protein by replacing it with alanine can result in the production of a YAP mutant that remains constantly active (Das et al. 2016). Considering the involvement of YAP in human cancers, the preserved sequence of YAP across mammals, and its impact on cell proliferation, growth, and viability, we proposed the idea that increasing the expression of YAP5SA in CHO cells could simultaneously enhance various cell attributes related to the production of foreign proteins. To investigate this matter, we introduced a mutant YAP, called YAP5SA, into EPO-CHO-K1 cells. After the transfection process, we assessed various parameters, including viable cell density, product titer, and specific cell productivity. The findings from our study indicate that enhancing the expression of YAP5SA leads to an enlargement in cell size, a decrease in apoptosis, and a notable enhancement of productivity. This study demonstrates that EPO-CHO cells overexpressing YAP5SA control the expression of multiple target genes involved in various processes, such as proliferation, survival, and mTOR activation. The mRNA levels of *CTGF* and *CYR61*, which are well-established targets of YAP (Zhao et al. 2022), showed a significant increase. These genes are known to be involved in important cellular processes such as apoptosis and cell division, and they have previously been used as indicators of YAP activity at the transcriptional level (Mugahid et al. 2020). The current study demonstrated a notable upregulation in the transcription of the *c-MYC* gene, a proto-oncogene responsible for encoding a transcription factor (Jaluria et al. 2007). This enhanced expression of *c-MYC* is associated with its ability to regulate diverse biological activities. Previous studies have demonstrated that *c-MYC* is capable of initiating cell cycle advancement, promoting cell proliferation (Cao et al. 2017; Ifandi and Al‐Rubeai 2005), and overseeing various stages of ribosome biogenesis, including the stimulation of ribosomal protein synthesis (Donati et al. 2012; Ifandi and Al‐Rubeai 2005; Mori et al. 2021). Supporting this theory, a study conducted on muscle satellite cells revealed that the constitutively active YAP leads to an upregulation of genes linked to the process of ribosome biogenesis and assembly (Judson et al. 2012). Cell proliferation is closely intertwined with cell-cycle progression. We compared the cell-cycle profiles between the EPO-CHO cell line overexpressing YAP5SA and the control EPO-CHO cells. We observed that the overexpression of YAP5SA facilitates the progression of the cell cycle by inducing the transition from the G1 to S phase (Fig. 6). This observation aligns with the observed profile of cell proliferation (Fig. 4). In previous studies conducted on CHO cultures, it was demonstrated that the percentage of cells in the S phase can be used to predict alterations in cell number and represents the dynamics of cell culture proliferation (Kuystermans and Al-Rubeai 2009). Therefore, it is plausible to propose that the enhanced growth rate observed in OE-YAP5SA cells results from the increased proportion of cells in the S phase. Previous studies have shown that c-Myc positively regulates the transcription of Cdc25A, which serves as a positive regulator of cdk2. Furthermore, c-Myc has been identified as a negative regulator of p27 (Donjerkovic and Scott 2000). P27 is classified as a cyclin-dependent kinase inhibitor (CKI) (Yang and Xu 2011) crucial in inducing G1 cell cycle arrest. It accomplishes this by binding to cyclin/CDK complexes, specifically cyclin E/CDK2, cyclin D/CDK2, and cyclin D/CDK4, all of which are necessary for the transition of cells from the G1 to S phase (Kumar et al. 2007). Based on these findings, it can be inferred that the observed cell cycle progression is likely attributed to the increased expression of *c-MYC*. Additionally, earlier research has identified an association between the mTOR and Hippo pathways, as an upregulation of mTOR has been shown to notably elevate both YAP mRNA and protein expression levels, leading to enhanced nuclear accumulation of YAP (Xu et al. 2020). Tumaneng et al. discovered that YAP plays a role in regulating the expression of the tumor suppressor *PTEN* and influences the PI3K/mTOR pathway, which governs cell size by controlling protein translation and autophagy. They showed that *PTEN*, which acts as a negative regulator of mTOR, plays a vital role in mediating the regulation of mTOR by YAP (Xu et al. 2021). In this study, the overexpression of YAP led to a decrease in the mRNA levels of the tumor suppressor *PTEN*. Correspondingly, the loss of PTEN due to the YAP overexpression led to an increase in mTOR expression, while the transcription level of *S6K* did not show any significant changes. Several studies have demonstrated that the overexpression of YAP leads to an elevation in the phosphorylation level of S6K, which serves as a direct substrate of mTOR (Tumaneng et al. 2012; Xu et al. 2021). Based on these findings, it can be concluded that the overexpression of YAP results in an increased phosphorylation of S6K. As c-Myc and mTOR are known to be powerful stimulators of ribosome biogenesis (Jiao et al. 2023), the upregulation of c-Myc expression and activation of mTOR induced by YAP could potentially contribute to the hypertrophic response. This contribution may be achieved by increasing the rate of protein synthesis through an enhancement in translational capacity.

Previous studies have indicated that YAP has the ability to interact with p73, a transcription factor associated with p53. This interaction leads to an augmentation of p73’s transcriptional activity (Basu et al. 2003). To investigate the potential involvement of YAP in p73-mediated signaling, we assessed the expression of *BAX*, a transcriptional objective of p73. Intriguingly, we show here that the upregulation of *BAX* expression was not attained through the transfection of EPO-CHO cells with the YAP5SA construct.

In the final phase, we examined the impact of YAP5SA overexpression on the overall and specific productivity of EPO. Remarkably, the overexpression of YAP5SA in EPO-CHO cells led to a threefold rise in titer compared to control cells, along with a 1.5-fold augmentation in specific productivity (QP). Hence, it is evident that the overexpression of YAP5SA not only enhances cell proliferation but also exhibits a substantial influence on the production of recombinant proteins within these cells.

This study conclusively demonstrated that introducing the mutant form of YAP resulted in significant and simultaneous improvements in various cell attributes relevant to producing heterologous proteins. These improvements encompassed cell growth, proliferation, viability, and productivity. While various transgenes have been utilized to enhance these cell characteristics individually, our study proposes that the overexpression of YAP5SA can effectively address multiple bioprocess-related limitations simultaneously.

In summary, based on our knowledge, this study represents one of the initial reports documenting the overexpression of the mutant form of YAP in CHO cells and investigating its impact on cell growth and the production of recombinant proteins. The findings of this study suggest that modifying CHO cells through the overexpression of YAP5SA effectively improves the productivity of these cells. This research indicates that YAP5SA holds promise as a potential candidate for improving CHO cells and enhancing the production of recombinant proteins. To validate its efficacy, it would be necessary to conduct extensive testing of YAP5SA with a broader selection of pharmaceutical recombinant proteins, including monoclonal antibodies, which are of significant interest. Additionally, conducting further experiments using high-produced CHO suspension cell lines would help determine the practicality of implementing YAP5SA in industrial settings.

## Supplementary Information

Below is the link to the electronic supplementary material.

Supplementary file1 (PDF 787 KB)

## Data Availability

The data generated during this study will be made available upon reasonable request.
