# Supplementary material for: Enhancing the productivity and proliferation of CHO-K1 cells by oncoprotein YAP (Yes-associated protein)
Source: Appl Microbiol Biotechnol. 2024 Apr 4;108:285. doi: 10.1007/s00253-024-13122-5 (PMC10994876; doi:10.1007/s00253-024-13122-5)
Supplement: Supplementary file 1 [file 253_2024_13122_MOESM1_ESM.pdf]

Supplementary Information

Journal name:

Applied Microbiology and Biotechnology

Manuscript title:

Enhancing the Productivity and Proliferation of CHO-K1 Cells by Oncoprotein YAP (Yes-Associated Protein)

The names of the authors:

Farnaz Roshanmehr[1,2], Shahriyar Abdoli[1,2], Zahra Bazi[2], Maryam Jari[1,2], Majid Shahbazi[1,3*]

The affiliation of authors:

[1] Medical Cellular & Molecular Research Center, Golestan University of Medical Sciences, Gorgan, Iran

[2] Department of Medical Biotechnology, School of Advanced Technologies in Medicine, Golestan University of Medical Sciences, Gorgan, Iran

[3]Arya Tina Gene (ATG), Biopharmaceutical Company, Gorgan, Iran

* Corresponding author:

Majid Shahbazi

E-mail: shahbazimajid@yahoo.co.uk, Shahbazim@atgbio.com

```
mYAP           1 MEPAQQPPPQPAPQGPAPP---------------SVSPAGTPAAPPAPPAGHQVVHVRGD 45
chYAP          1 MEPAQQPPPQPAPQGPVPPSA-------------PAPPSGTPAAPPAPPAGHQVVHVRGD 47
hYAP           1 MDPGQQPPPQPAPQGQGQPPSQPPQGQGPPSGPGQPAPAATQAAPQAPPAGHQIVHVRGD 60
                 *:*.***********   *                 .*:.* *** *******:******

mYAP          46 SETDLEALFNAVMNPKTANVPQTVPMRLRKLPDSFFKPPEPKSHSRQASTDAGTAGALTP 105
chYAP         48 SETDLEALFNAVMNPKTANVPQTVPMRLRKLPDSFFKPPEPKSHSRQASTDAGTAGALTP 107
hYAP          61 SETDLEALFNAVMNPKTANVPQTVPMRLRKLPDSFFKPPEPKSHSRQASTDAGTAGALTP 120
                 ************************************************************

mYAP         106 QHVRAHSSPASLQLGAVSPGTLTASGVVSGPAAAPAAQHLRQSSFEIPDDVPLPAGWEMA 165
chYAP        108 QHVRAHSSPASLQLGAVSPGTLTPT-VVSGPAATPAAQHLRQSSFEIPDDVPLPAGWEMA 166
hYAP         121 QHVRAHSSPASLQLGAVSPGTLTPTGVVSGPAATPTAQHLRQSSFEIPDDVPLPAGWEMA 180
                 ***********************.: *******:*:************************

mYAP         166 KTSSGQRYFLNHNDQTTTWQDPRKAMLSQLNVPAPASPAVPQTLMNSASGPLPDGWEQAM 225
chYAP        167 KTSSGQRYFLNHIDQTTTWQDPRKAMLSQLNVPTPASTAVPQTLMNSASGPLPDGWEQAM 226
hYAP         181 KTSSGQRYFLNHIDQTTTWQDPRKAMLSQMNVTAPTSPPVQQNMMNSASGPLPDGWEQAM 240
                 ************ ****************:**.:*:*..* *.:****************

mYAP         226 TQDGEVYYINHKNKTTSWLDPRLDPRFAMNQRITQSAPVKQPPPLAPQSPQGGVLGGGSS 285
chYAP        227 TQDGDVYYINHKNKTTSWLDPRLDPRFAMNQRITQSAPVKQPPPLAPQSPQGGVLGGGNS 286
hYAP         241 TQDGEIYYINHKNKTTSWLDPRLDPRFAMNQRISQSAPVKQPPPLAPQSPQGGVMGGSNS 300
                 ****::***************************:********************:**..*

mYAP         286 NQQQQIQLQQLQMEKERLRLKQQELFRQELALRSQLPTLEQDGGTPNAVSSPGMSQELRT 345
chYAP        287 SQQQQMQLQQLQMEKERLRLKQQELLRQELALRSQLPTLEQDGGTQNAVSSPGMSQELRT 346
hYAP         301 NQQQQMRLQQLQMEKERLRLKQQELLRQELALRSQLPTLEQDGGTQNPVSSPGMSQELRT 360
                 .****::******************:******************* *.************

mYAP         346 MTTNSSDPFLNSGTYHSRDESTDSGLSMSSYSIPRTPDDFLNSVDEMDTGDTISQSTLPS 405
chYAP        347 MTTNSSDPFLNSGTYHSRDESTDSGLSMSSYSVPRTPDDFLNSVDEMDTGDTINQSTLPS 406
hYAP         361 MTTNSSDPFLNSGTYHSRDESTDSGLSMSSYSVPRTPDDFLNSVDEMDTGDTINQSTLPS 420
                 ********************************:********************.******

mYAP         406 QQSRFPDYLEALPGTNVDLGTLEGDAMNIEGEELMPSLQEALSSEIL-DVESVLAATKLD 464
chYAP        407 QQSRFPDYLEAIPGTNVDLGTLEGDAMNIEGEELMPSLQEALSSDILNDMESVLAATKLD 466
hYAP         421 QQNRFPDYLEAIPGTNVDLGTLEGDGMNIEGEELMPSLQEALSSDILNDMESVLAATKLD 480
                 **.********:*************.******************:** *:**********

mYAP         465 KESFLTWL 472
chYAP        467 KESFLTWL 474
hYAP         481 KESFLTWL 488
                 ********
```

Fig. S1 Amino acid sequence alignment of YAP from various organisms, including human, mouse, and Chinese hamster, for the identification of the consensus phosphorylation sequence for LATS. The LATS consensus phosphorylation sites are highlighted.

ATGGAGCCCGCGCAGCAGCCGCCGCCCCAGCCGGCCCCGCAAGGCCCCGTGCCGCCGTCCGCGCCCGCGC

CGCCGTCAGGGACCCCCGCGGCCCCGCCCGCGCCCCCGGCCGGCCACCAAGTCGTGCACGTCCGCGGAGA

C**GCG**GAGACCGACCTGGAGGCGCTCTTCAACGCCGTCATGAACCCCAAGACGGCCAACGTGCCGCAGACC

GTGCCCATGCGGCTGCGCAAGCTGCCCGACTCCTTCTTCAAGCCGCCCGAGCCCAAGTCCCACTCGCGAC

AGGCT**GCT**ACTGATGCAGGCACTGCAGGAGCTCTGACTCCACAGCACGTTCGGGCTCAC**GCCGCT**CCAGC

A**GCC**CTGCAGCTGGGAGCCGTTTCTCCTGGGACTCTCACCCCTACTGTGGTGTCTGGTCCTGCAGCCACC

CCTGCAGCACAGCATCTCCGACAG**GCTGCC**TTTGAGATCCCTGATGATGTACCCCTGCCTGCAGGCTGGG

AGATGGCCAAGACATCGTCTGGTCAGAGATACTTCTTAAATCACATCGATCAGACAACAACATGGCAGGA

CCCCAGGAAGGCCATGCTCTCCCAACTGAATGTCCCTACGCCTGCCAGCACAGCAGTGCCTCAGACCCTG

ATGAACTCCGCCTCAGGACCTCTTCCTGATGGATGGGAGCAAGCCATGACGCAGGATGGAGATGTGTACT

ACATAAACCATAAGAACAAGACTACGTCCTGGCTGGACCCAAGGCTCGACCCTCGTTTTGCTATGAACCA

GAGAATCACTCAGAGCGCTCCAGTGAAGCAGCCGCCACCCTTGGCTCCCCAGAGCCCACAGGGAGGTGTC

CTGGGTGGTGGCAACTCCAGCCAGCAGCAGCAGATGCAACTGCAGCAGCTCCAGATGGAGAAGGAGAGAC

TGCGGTTGAAACAGCAAGAATTACTGCGGCAGGAATTAGCTCTCCGAAGCCAGTTGCCCACACTGGAGCA

GGACGGAGGAACTCAGAATGCTGTGTCTTCCCCCGGGATGTCTCAGGAATTGAGGACAATGACAACCAAT

AGTTCAGATCCCTTTCTTAACAGTGGCACCTATCACTCTAGAGATGAG**GCC**ACGGACAGTGGCCTCAGCA

TGAGTAGCTACAGTGTCCCTCGGACCCCGGATGACTTCCTGAACAGTGTTGATGAAATGGATACAGGTGA

CACTATCAACCAAAGCACCCTGCCTTCACAGCAGAGCCGTTTCCCAGATTACCTTGAAGCCATCCCTGGG

ACAAATGTGGACCTTGGAACACTGGAAGGAGATGCGATGAACATAGAAGGAGAGGAGCTGATGCCCAGCC

TGCAGGAAGCACTGAGTTCCGACATCCTTAATGACATGGAGTCTGTGTTGGCTGCCACCAAGCTAGATAA

AGAAAGCTTTCTCACATGGTTATAG

Fig. S2 Nucleotide sequence encoding the YAP-5SA which is inserted within the PB513B-1 plasmid. Letters in red indicate bases changed to create the codon for alanine, resulting in the substitution of the serine codon with alanine, shown in highlighted color.
